# Contribution of KCNQ and TREK Channels to the Resting Membrane Potential in Sympathetic Neurons at Physiological Temperature

**DOI:** 10.3390/ijms21165796

**Published:** 2020-08-12

**Authors:** Paula Rivas-Ramírez, Antonio Reboreda, Lola Rueda-Ruzafa, Salvador Herrera-Pérez, Jose Antonio Lamas

**Affiliations:** 1 Department of Functional Biology and Health Sciences, Faculty of Biology-CINBIO-IBIV, University of Vigo, Campus Lagoas-Marcosende, 36310 Vigo, Spain; privas84@hotmail.com (P.R.-R.); lolarrzg@gmail.com (L.R.-R.); ssalva4@me.com (S.H.-P.); 2 Functional Architecture of Memory Department, Leibniz-Institute for Neurobiology, 39118 Magdeburg, Germany

**Keywords:** resting membrane potential, perforated patch, sympathetic neurons, TREK current, M-current

## Abstract

The ionic mechanisms controlling the resting membrane potential (RMP) in superior cervical ganglion (SCG) neurons have been widely studied and the M-current (I_M_, KCNQ) is one of the key players. Recently, with the discovery of the presence of functional TREK-2 (TWIK-related K^+^ channel 2) channels in SCG neurons, another potential main contributor for setting the value of the resting membrane potential has appeared. In the present work, we quantified the contribution of TREK-2 channels to the resting membrane potential at physiological temperature and studied its role in excitability using patch-clamp techniques. In the process we have discovered that TREK-2 channels are sensitive to the classic M-current blockers linopirdine and XE991 (IC_50_ = 0.310 ± 0.06 µM and 0.044 ± 0.013 µM, respectively). An increase from room temperature (23 °C) to physiological temperature (37 °C) enhanced both I_M_ and TREK-2 currents. Likewise, inhibition of I_M_ by tetraethylammonium (TEA) and TREK-2 current by XE991 depolarized the RMP at room and physiological temperatures. Temperature rise also enhanced adaptation in SCG neurons which was reduced due to TREK-2 and I_M_ inhibition by XE991 application. In summary, TREK-2 and M currents contribute to the resting membrane potential and excitability at room and physiological temperature in the primary culture of mouse SCG neurons.

## 1. Introduction

Traditionally, superior cervical ganglion neurons (SCG) have been used as a model to study the resting membrane potential (RMP) and excitability [1,2]. The RMP in rodent sympathetic neurons is sustained around −60 mV thanks to the contribution of voltage-dependent and independent currents. Considering the voltage-dependent currents, the main contributors to the RMP in SCG neurons are the potassium M-current (I_M_), the cationic h-current (I_h_) and a sodium persistent current (I_NaP_). All of them are activated at RMP values and do not inactivate or their inactivation is very slow [3]. I_M_ tends to hyperpolarize while I_h_ tends to depolarize the membrane potential in a similar amount (≈ 7 mV), having a strong stabilizing effect on the RMP [4]. Furthermore, I_NaP_ contributes a depolarizing component to the RMP [5]. The voltage independent currents involved in maintaining the RMP in SCG neurons are carried by the Na/K pump (leak-like) and by two pore domain potassium channels (K2P), both of them contributing a hyperpolarizing factor [4,6].

The K2P family [7] is distributed into six subfamilies according to their physical and chemical properties in TWIK (Tandem of pore-domains in a Weakly Inward rectifying K^+^ channel), THIK (Tandem pore-domain Halothane Inhibited K^+^ channel), TASK (TWIK-related acid-sensitive K^+^ channel), TALK (TWIK-related alkaline pH-activated K^+^ channel), TRESK (TWIK-related spinal cord K^+^ channel) and TREK. In mouse SCG neurons, TRESK and TREK subfamilies are the best expressed K2P channels and within the TREK subfamily, TREK-2 channels are the most expressed, followed by TREK-1 and TRAAK (TWIK-related arachidonic acid-stimulated K^+^ channel) [8]. While the K2P channels are active at all membrane potentials and hence are considered voltage independent [9], I_M_ is clearly voltage dependent with a threshold around −60 mV [1,10] and a maximum activation at −30 mV [11].

Often, the depolarization of the RMP and the reduction of the adaptation induced by the muscarinic agonist oxotremorine-M (oxo-M), and by nootropic drugs like linopirdine and its analog XE991, are attributed to I_M_ inhibition [10,12]. Nevertheless, we have seen recently that TREK-2 channels in mouse SCG neurons are also inhibited by the muscarinic agonist oxo-M through G-proteins and a second messenger cascade [13]. Moreover, inhibition of TREK-2 current [14] and M-current [15] by bradykinin hormone depolarizes the RMP. On the other hand, the inhibition of TREK currents with fluoxetine [16] produced a depolarization of the membrane potential and reduced the latency to the first evoked action potential, without affecting adaptation [6] at room temperature.

It is a general observation that TREK channels show weak activity at room temperature and that their open probability increases when the temperature is raised [17,18,19,20]. Hence, we hypothesized that the importance of this current on the membrane potential should be greatly potentiated at physiological temperature. Importantly, the conductance of I_M_ is also enhanced by increasing temperature [21].

In this study we aimed to quantify the importance of the TREK and M currents on the maintenance of the resting membrane potential and excitability of the sympathetic neurons. Using channel blockers (mainly tetraethylammonium (TEA), linopirdine and XE991) to separate the contribution of each current, we show that they contribute equally to keeping the resting membrane potential at physiological temperatures.

## 2. Results

### 2.1. Linopirdine and Its Analog XE991 Inhibit TREK-2 Current

Within the K2P family, only the TREK subfamily is activated by the neuroprotective agent riluzole (2-amino-6-trifluoromethoxy benzothiazole) [6,22,23]. With the final objective of discriminating between the contribution of the TREK-2 and M-current to the resting membrane potential we needed a selective inhibitor for at least one of them. Consequently, we decided to demonstrate that traditional M-current blockers, linopirdine and XE991, did not affect the TREK-2 current activated by riluzole (I_RIL_) [6]. Surprisingly we found that both substances strongly inhibited I_RIL_, not only in mouse SCG neurons (TREK-2 native current) but also in a HEK293 cell line (TREK-2 expressed currents).

**Figure 1:**
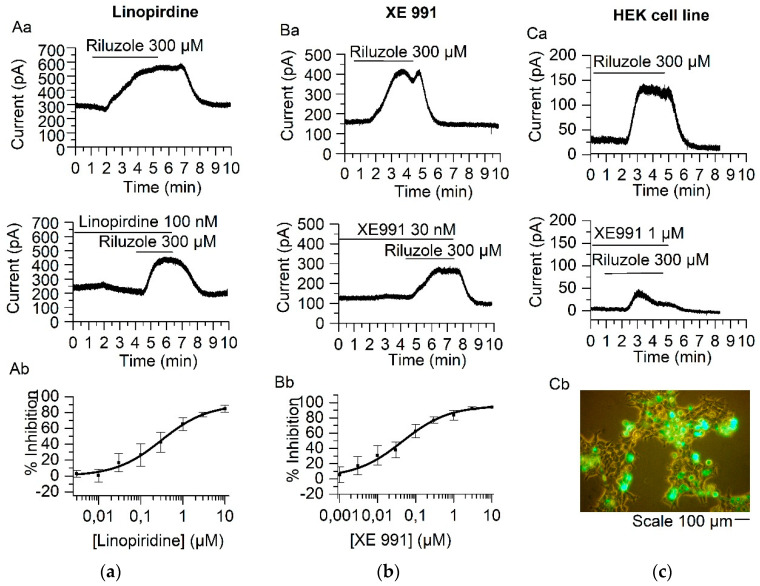
Inhibition of I_RIL_ by linopirdine and XE991 in SCG (superior cervical ganglion) neurons and in HEK293. (**A**) Inhibition of I_RIL_ by linopirdine 100 nM (concentration close to the IC50; (**Aa**)). Dose–response curve for the inhibition of I_RIL_ by linopirdine (concentrations (µM) = 0.003, 0.01, 0.03, 0.1, 0.3, 1, 3, 10) showing an IC_50_ of 0.310 ± 0.06 µM (**Ab**). (**B**) Inhibition of I_RIL_ by XE991 30 nM (concentration close IC_50_; (**Ba**)). Dose–response curve for I_RIL_ inhibited by XE991 (concentrations (µM) = 0.001, 0.003, 0.01, 0.03, 0.1, 0.3, 1, 3, 10) resulting in an IC_50_ of 0.044 ± 0.013 µM (**Bb**). (**C**) The riluzole-evoked current in HEK293 cells was strongly suppressed by 1 µM XE991 (**Ca**). HEK293 cells transfected with TREK-2 channels (**Cb**).

Mouse SCG neurons were treated with the cocktail (TEA, TTX and Cs^+^) and riluzole (300 µM) was then applied in the bath solution at room temperature. Riluzole-activated currents were recorded using the whole-cell patch-clamp technique in the perforated-patch variant. The double application of riluzole, first as control and second in presence of linopirdine, showed considerable I_RIL_ inhibition (Figure 1Aa). Application of different concentrations of linopirdine showed a dose–response curve with an IC_50_ of 0.310 ± 0.06 µM, a maximum inhibition of 84.89 ± 4.29% at 10 µM and a Hill coefficient of 0.78 ± 0.12, (Figure 1Ab, *n* = 16). The linopirdine analog, XE991, also inhibited I_RIL_ (Figure 1Ba) in mouse SCG neurons, showing a dose–response curve with an IC_50_ of 0.044 ± 0.013 µM; the maximum inhibition was 92.48 ± 2.33% at 3 µM of XE991 and the Hill coefficient was 0.71 ± 0.09 (Figure 1Bb, *n* = 10).

As expected, TREK-2 channels expressed in HEK293 cells with a GFP marker (Figure 1Cb) were strongly blocked by XE991 application: 110.35 ± 25.36 pA to 27.91 ± 4.55 pA (inhibition 73.85 ± 1.93%, *p* < 0.05, *n* = 3, Figure 1Ca). Likewise, application of linopirdine (300 nM) reduced the heterologous expressed current from 189.49 ± 60.20 pA to 69.22 ± 28.95 pA (inhibition 65.46 ± 5.93%, *p* < 0.05, *n* = 4).

**Figure 2:**
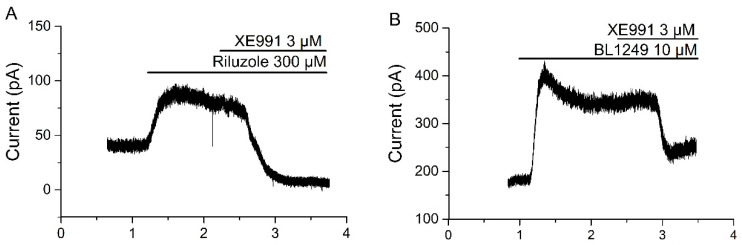
XE991 inhibits riluzole- and BL1249-activated currents in SCG (superior cervical ganglion) cells recorded in voltage-clamp; (**A**) XE991 (3µM) was applied in the bath solution when I_RIL_ is maximal (125.47 ± 52.93 pA, *n* = 4). One minute later, XE991 provoked an inward current (−121.66 ± 66.87 pA, *n* = 4); (**B**) XE991 (3µM) was applied in the bath solution when TREK current activator BL1249 (10µM) provoked the maximal current (82.41 ± 27.07 pA; *n* = 4). XE991 generated an inward current of −67.26 ± 18.73 pA (*n* = 4).

In addition, XE991 was applied upon the maximal effect of riluzole in SCG neurons in order to corroborate the effect of XE991 directly on riluzole-activated current. As reported before, activation of TREK-1 and TREK-2 currents by riluzole is often biphasic and the activation is transient [22]; however, the effect of XE991 (3 µM) was so strong that it was easily measured (Figure 2A). In our hands riluzole induced an outward current of 125.47 ± 52.93 pA (I_RIL_, *n* = 4) and we considered the XE991 inhibitory effect as an inward current (I_XE991_ = −121.66 ± 66.87 pA, *n* = 4). Similar experiments were designed using another TREK current activator, BL1249 [24]. The application of BL1249 10µM provoked an outward current of 82.41 ± 27.07 pA (I_BL_; *n* = 4). On the maximal effect of BL1249, we applied XE991 (3µM) that provoked an inward current of −67.26 ± 18.73 pA (*n* = 4), Figure 2B.

### 2.2. I_M_ and TREK-2 Currents Are Enhanced by Temperature

We have previously demonstrated that TREK-2 currents activated by riluzole (I_RIL_) are insensitive to TEA [6,25,26] and that I_RIL_ and I_M_ are different currents [13]. Taking advantage of the fact that I_M_ is inhibited by TEA [1,27], the current inhibited by TEA (15 mM), called I_TEA_, should be mainly M-current. Applying the same logic, we tested XE991 (3 µM) in the presence of TEA. The current sensitive to XE991 should be carried mainly through TREK-2 channels and it was nominated I_XE991_. These voltage-clamp experiments (whole-cell perforated-patch) were carried out in the presence of TTX and CsCl as they do not affect any of the currents.

**Figure 3:**
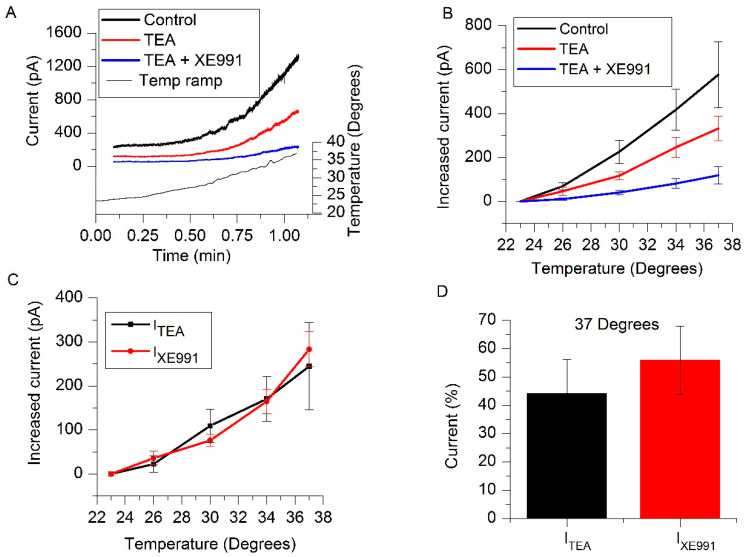
Increase of I_TEA_ and I_XE991_ by temperature rise in SCG (superior cervical ganglion) neurons. (**A**) Example of current increases in control (black trace), in presence of TEA (15 mM, red trace) and in the presence of TEA + XE991 (3 µM) (blue trace), produced by a temperature rise at 0.23 °C/s (black thin trace) from 23 °C to 37 °C in one SCG neuron. (**B**) Increase of the same currents when the temperature was raised from 23 °C to different temperatures (26, 30, 34 and 37 °C; *n* = 5). (**C**) Variations of the subtracted I_TEA_ (Control-TEA) and I_XE991_ (TEA-(TEA + XE991)) currents with temperature (*p* = 0.29, *n* = 5). (**D**) Percentages of I_TEA_ and I_XE991_ activated at 37 °C were 44.19 ± 12.07% and 55.81 ± 12.07% respectively.

TREK channels have a low activity at room temperature and this activity is greatly enhanced by increasing it [18,19,20]. In order to magnify TREK-2 currents and to highlight its contribution to the RMP, we heated the bath solution from room (23 °C) to physiological temperature (37 °C) at a rate of 0.23 °C/s (Figure 3A) and measured the outward current (fixing the voltage at −30 mV) in control (CsCl + TTX) and in the presence of TEA and TEA + XE991. Figure 3B shows the increase of the currents at different temperatures and the current at 23 °C was considered zero for any treatment. This protocol allowed us to easily obtain the current sensitive to TEA (I_TEA_ ≈ I_M_) and to XE991 (I_XE991_ ≈ I_TREK_) separately by subtraction (Figure 3C). The rate of current change for every 10 °C of temperature increase (Q10) was 7.32 ± 1.97 for I_TEA_ and 18.11 ± 10.67 for I_XE991_. The increase in temperature seems to affect both currents similarly (Figure 3C; Q10 comparison: *p* = 0.29, *n* = 5) and the contribution of them to the total current is also similar at any temperature. Figure 3D shows their contribution at 37 °C (I_TEA_ = 44.19 ± 12.07% and I_XE991_ = 55.81 ± 12.07%, *p* = 0.68, *n* = 5). The temperature increases from room to physiological temperature provoked a decrease of 0.1 in pH in the bath solution. The extracellular acidification of about 0.1 in pH scarcely increased the TREK-2 current [28].

### 2.3. Contribution of I_M_ and I_TREK_ to the RMP at Physiological Temperature

The objective of these experiments was to quantify the involvement of I_M_ and I_TREK_ on the RMP of SCG neurons at room and physiological temperatures. We used a current-clamp (bridge mode) protocol with the membrane potential manually fixed at −60 mV (close to the RMP of SCG neurons [10]) and a bath containing TTX (0.5 µM) and CsCl (1 mM).

**Figure 4:**
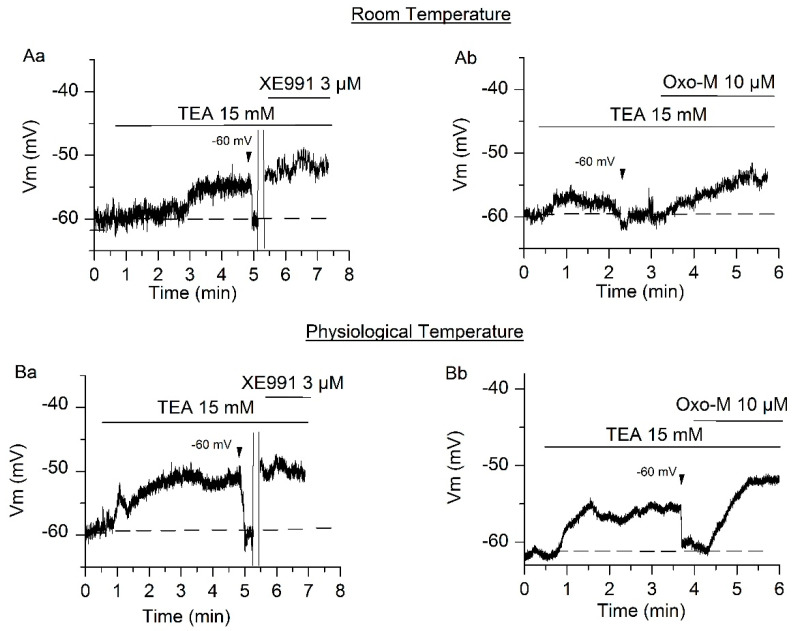
I_M_ and I_TREK_ implication on the RMP (resting membrane potential) in SCG (superior cervical ganglion) neurons. (**A**) The bath application of I_M_ blocker TEA provoked a depolarization of 4.58 ± 1.12 mV on SCG neurons, fixed at −60 mV in current-clamp mode, at room temperature. Posterior application of XE991 (**Aa**) and oxo-M (**Ab**) on neurons fixed at −60 mV depolarized the membrane potential 5.8 ± 1.32 mV and 7.14 ± 0.85 mV, respectively. (**B**) The same experiment was performed at physiological temperature (37 °C) provoking a depolarization of 5.99 ± 0.73 mV by TEA (**Ba**,**Bb**), 7.45 ± 1.99 mV by XE991 (**Ba**) and 9.29 ± 1.13 mV by oxo-M (**Bb**). The arrowhead indicates that membrane potential was fixed manually to −60 mV after TEA effect. The double bar indicates XE991 application in voltage-clamp mode on neurons fixed at −30 mV.

First, changes in the membrane potential value were observed at room temperature (23 °C). In these conditions, the application of TEA (15 mM) provoked a depolarization (from −60.33 ± 0.27 mV to −55.75 ± 0.55 mV) of 4.58 ± 1.12 mV (*p* < 0.01, *n* = 10, Figure 4Aa), ascribed to the inhibition of the M-current. Still in the presence of TEA, the membrane was again manually settled at −60 mV (Figure 4Aa, arrowhead) and the amplifier was turned to the voltage-clamp mode in order to apply XE991 (3 µM) at −30 mV and to avoid the possible voltage-dependent effect of XE991 [10]. The amplifier was returned to bridge mode when the effect of the XE991 was full and stable and we observed a depolarization of 5.8 ± 1.32 mV (*p* < 0.01, *n* = 12), from −60.16 ± 0.35 mV to −54.36 ± 0.23 mV, that we attributed to the inhibition of TREK-2 channels (Figure 4Aa).

We have recently shown that TREK-2 currents are inhibited by muscarinic agonists reducing PIP_2_ [13]. At room temperature and in the presence of the blocking mixture TTX, Cs and TEA, application of 10 µM oxo-M (oxotremorine methiodide, a nonselective muscarinic acetylcholine receptor agonist often working through the PLC pathway) produced a significant depolarization of 7.14 ± 0.85 mV from −59.21 ± 0.52 mV to −52.07 ± 0.46 mV (*p* < 0.001, *n* = 7, Figure 4Ab).

Similar experiments were performed at physiological temperature (37 °C). In this case, the application of TEA (15 mM) provoked a depolarization of 5.99 ± 0.73 mV (*p* < 0.001, *n* = 13, Figure 4B). Still in the presence of TEA, the application of XE991 (3 µM) caused a depolarization of 7.45 ± 1.99 mV (*p* < 0.05, *n* = 4, Figure 4Ba), while the application of oxo-M in the same conditions depolarized 9.29 ± 1.13 mV (*p* < 0.001, *n* = 7, Figure 4Bb).

### 2.4. The Increase in Temperature Enhances Adaptation

It is generally accepted that the characteristic spike frequency adaptation (SFA) presented by SCG neurons is mainly under M-current control at room temperature [6,10]. We have previously shown that at this temperature, the inhibition of TREK channels by fluoxetine did not affect the number of action potentials induced by depolarizing current-steps, but reduced the latency to the first action potential [6]. Because TREK channels are very sensitive to the temperature, we wondered whether these channels could have a more prominent role in SFA at physiological temperatures.

**Figure 5:**
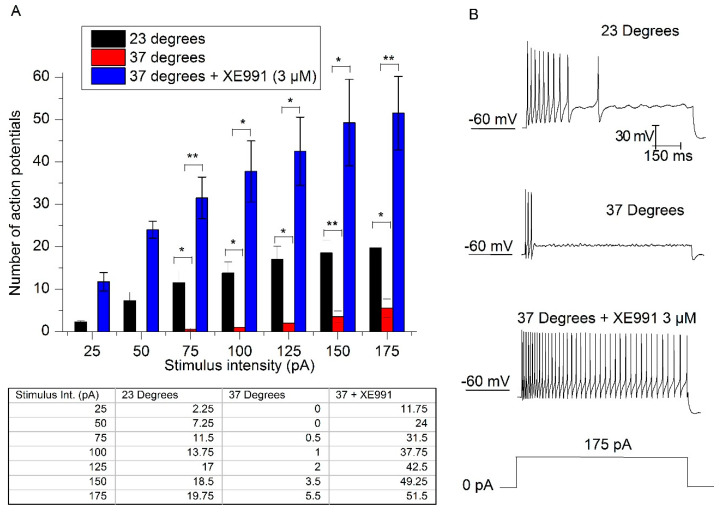
XE991 reduces adaptation at physiological temperatures in SCG (superior cervical ganglion) neurons. (**A**) Number of action potentials in response to different intensities at 23 °C (black bars), 37 °C (red bars) and 37 °C with XE991 (blue bars), (* *p* < 0.05, ** *p* < 0.01, *n* = 5). (**B**) Spike firing in response to 175 pA current-injection at 23 °C, 37 °C and 37 °C with XE991. The membrane potential was manually settled to −60 mV. The table shows the number of action potentials at different currents injected at 23 °C, 37 °C and 37 °C with XE991.

In bridge-mode we applied depolarizing 1 s current injections of increasing amplitude (from −50 to 175 pA in 25 pA steps) with the voltage of the membrane manually settled at −60 mV. The adaptation (quantified as the number of action potentials fired in response to depolarizing current injections) observed at room temperature was strongly increased when the bath was heated to 37 °C (Figure 5A,B). The combined inhibition of M and TREK currents by XE991 dramatically reduced adaptation and provoked a strong increase in the number of action potentials fired in one second (Figure 5B, bottom trace). To apply XE991 (3 µM) we switched the amplifier to voltage-clamp mode and fixed the membrane potential at −30 mV to overcome XE991 voltage-dependency [10].

### 2.5. Temperature Modifies the Action Potential Frequency and Latency

**Figure 6:**
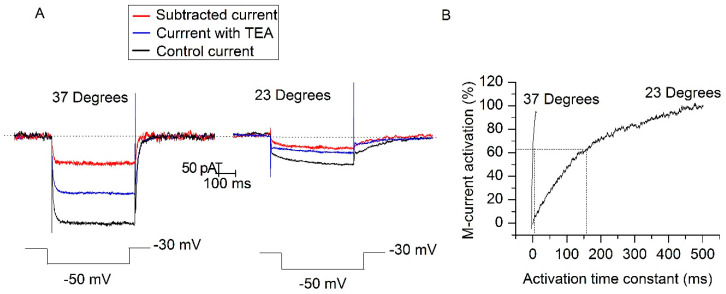
Opening and closing time constants for I_TEA_ in SCG (superior cervical ganglion) neurons. (**A**) Voltage-steps from −30 to −50 mV allowed us to first deactivate and then activate the M-current. The current in the presence of TEA (15 mM) (black trace) was subtracted from the control (blue trace) to obtain I_TEA_ (red trace). (**B**) The activation time constant for I_TEA_ decreased from 157.43 ± 12.62 to 7.18 ± 1.90 ms when the temperature was increased from 23 to 37 °C (*n* = 9).

M-current is characterized by rather slow activation and deactivation time constants (τ) at room temperature [11,29,30]. For that, the contribution of the M-current to the behavior of the neuron should be small during the first milliseconds of depolarizing current injection. On the contrary, TREK-2 channels should be operative from the start of the injection as they are essentially voltage-independent [31]. The difference between both channels’ activation time should be useful to isolate TREK-2 current, at least at room temperature. For that, first we determined the activation and deactivation time constants of the TEA sensitive current (mainly M-current) with the classic voltage-clamp protocol used to study M-current. The membrane potential was fixed at −30 mV and stepped to −50 mV for 500 ms in the presence of CsCl + TTX (Figure 6A). The current sensitive to TEA (15 mM) was analyzed both at physiological (Figure 6A, left) and at room temperatures (Figure 6A, right). The decrease of temperature (Figure 6A, right) significantly increased the deactivation time constants (τ_off_) from 8.26 ± 1.88 to 67.61 ± 5.16 ms (*p* < 0.001, *n* = 9) and opening time constant (τ_on_) from 7.18 ± 1.90 to 157.43 ± 12.62 ms (*p* < 0.001, *n* = 9, Figure 6B). The high activation speed of I_TEA_ indicates that at 37 °C the M-current may participate in the firing characteristics from the beginning of the depolarizing current injection.

**Figure 7:**
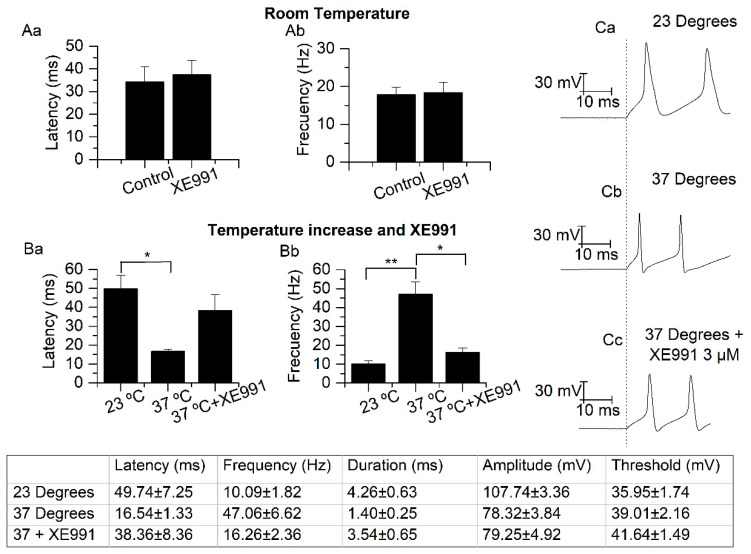
Effect of temperature and XE991 on the action potential parameters in SCG (superior cervical ganglion) neurons. (**A**) Latency (**Aa**) and frequency (**Ab**) do not change with the application of XE991 (3 µM, *n* = 7) at room temperature. (**B**) The latency to the first action potential decreased significantly (*p* < 0.05, *n* = 5) with the increases of temperature. Posterior application of XE991 (3 µM) provoked no significant change (*p* = 0.072, *n* = 5) (**Ba**). On the contrary, the early spike frequency was significantly increased (*p* < 0.01, *n* = 7) with increases of temperature and this increase was countervailed with XE991 (3 µM, *p* < 0.05, *n* = 5) (**Bb**) (* *p* < 0.05, ** *p* < 0.01). (**C**) Representative traces of spike firing in response to 175 pA current-injection at 23 °C (**Ca**), 37 °C (**Cb**) and 37 °C with XE991 (**Cc**) and the membrane potential fixed at −60 mV. The table shows latency, frequency of two first spikes, spike duration, amplitude and threshold at the first spike in response to 175 pA current-injection at 23 °C, 37 °C and 37 °C with XE991.

Surprisingly, the inhibition of M and TREK currents, by XE991 (3 µM) at room temperature, did not affect either the latency of the first action potential or the frequency measured using the two first action potentials (both measured in the first trace generating two or more action potentials). The results show that there are no differences between the frequency in absence (17.85 ± 1.93 Hz) and in presence (18.37 ± 2.68 Hz) of XE991 (*p* = 0.672, *n* = 7). The same was true for the latency in absence (34.19 ± 6.72 ms) and in presence (37.39 ± 6.32 ms) of XE991 (*p* = 0.535, *n* = 7) (Figure 7A). As the activation time constant for the M-current was about 157 ms (Figure 6B) and the latency and frequency were measured in the first 60 ms, the M current should be mostly inactive. This experiment indicated that the inhibition of TREK currents by XE991 did not affect the latency or the early action potential frequency at room temperature.

It was described above that the increase in temperature dramatically decreased the activation time constant of the M-current and increased the size of both M and TREK currents. The latency to the first action potential was clearly reduced from 49.74 ± 7.25 ms to 16.54 ± 1.33 ms (*p* < 0.05, *n* = 5) and the early frequency increased from 10.09 ± 1.82 Hz to 47.06 ± 6.62 Hz (*p* < 0.01, *n* = 7) when the temperature was increased from 23 to 37 °C (Figure 7B). The application of XE991 (3 µM) at physiological temperature to inhibit both currents, provoked no significant changes in the latency to 38.36 ± 8.36 ms (*p* < 0.05, *n* = 5), and in frequency induced a significant decrease to 16.26 ± 2.35 Hz (*p* < 0.05, *n* = 5). Moreover, we observed that the increase of temperature, from room to physiological temperature, decreased the spike duration (from 4.26 ± 0.65 to 1.40 ± 0.25 ms, *p* < 0.01, *n* = 5) and the amplitude (from 107.74 ± 3.36 to 78.32 ± 3.84 mV, *p* < 0.001, *n* = 5), without modifying the threshold (from 35.95 ± 1.74 to 39.01 ± 2.16 mV, *p* > 0.05, *n* = 5) in the first spike observed. The application of XE991 (3 µM) at physiological temperature recovered the spike duration (from 1.40 ± 0.25 to 3.54 ± 0.65 ms, *p* < 0.01, *n* = 5,) but did not modify the amplitude (from 78.32 ± 3.84 to 79.25 ± 4.92 mV, *p* > 0.05, *n* = 5) or the threshold (from 39.01 ± 2.16 to 41.64 ± 1.49 mV, *p* > 0.05, *n* = 5) in the first spike.

## 3. Discussion

Potassium currents are strongly implicated in maintaining the RMP and in regulating the excitability of neurons and other cell types. In SCG neurons, the involvement of I_M_ in RMP and spike frequency adaptation has been deeply characterized [1,4,10,32]). It is well known that RMP changes when I_M_ is modulated through GPCRs by muscarinic, bradykinin, purinergic and angiotensin II agonists [10,32,33] or directly by blockers such as linopirdine and XE991 [10,29] in SCG neurons. In 1996, the first member of the K2P family was described [7] and this potassium channel family is also involved in the maintenance of the RMP and excitability [14,34]. Because TREK channels are very sensitive to temperature [35], the main objective of this work was to determine the contribution of each of these two currents (M and TREK) to the resting membrane potential at physiological temperatures.

We took advantage of the fact that in the K2P family only the TREK subfamily is activated by the neuroprotective agent riluzole (2-amino-6-trifluoromethoxy benzothiazole) [6,22,23]. On the other hand I_M_, but not the TREK-2 current, is inhibited by tetraethylammonium (TEA) [1,25,26]. In fact, in our laboratory we have demonstrated that the TREK-2 current activated by riluzole (I_RIL_) is insensitive to TEA [6]. The activation curve of the TEA-sensitive current (I_TEA_), which is mostly carried by I_M_, clearly differs from that shown by I_RIL_, demonstrating that I_RIL_ and I_TEA_ are two different currents [13]. Our results show that both currents are similarly enhanced by temperature resulting in a clear increase in adaptation. Moreover, the contribution of the TREK-2 current to the RMP is similar to that of I_M_ at physiological temperature. The fact that M-current blockers (linopirdine and XE991) also blocked TREK-2 currents, in SCG neurons and HEK293 cells expressing TREK-2 channels, was both unexpected and relevant.

### 3.1. Isolation of I_M_ and TREK-2 Current

TREK channel activity at room temperature is strongly reduced and this fact is very significant since most of the studies in SCG neurons are performed at room temperature [4,10,36,37,38]. Compared to the other K2P members, the TREK subfamily shows a stronger activation by increasing temperature [19,20,39] and we hypothesized that using physiological temperatures could help us to better identify the roles of these currents in SCG neurons. On the other hand we know that only the TREK subfamily is activated by the neuroprotector riluzole [22,23] and that TEA does not affect TREK-2 currents [6,13] while it blocks I_M_ by about 70% at 15 mM [40,41].

However, application of TEA can also affect other currents such as the inward rectifier potassium current [42,43], transient potassium current (A-type) [43,44] or calcium-activated potassium current [44]. To avoid those “side effects”, linopirdine and its analog XE991 have been lately used to study I_M_ [12,29,45]. The application of these M-current blockers depolarizes the RMP [10] and changes the firing pattern [12]. Nevertheless, to our surprise, both linopirdine and XE991 block I_RIL_ in SCG neurons and TREK-2 channels expressed in HEK293 cells. In fact, TREK-2 current is more sensitive to linopirdine and XE991 (IC_50_ = 0.31 µM and IC_50_ = 0.045 µM, respectively) than I_M_ (IC_50_ = 2.56 µM and IC_50_ = 0.26 µM) in SCG neurons [10].

We attempted to isolate TREK-2 from I_M_ based on different temperature sensitivity, but we observed a similar activation of these currents when increasing temperature. According to Kang and colleagues [20], TREK-2 current increases sharply by temperature increase in cerebellar granule ganglion and dorsal root ganglion neurons (about 14 times per 10 degrees). I_M_ temperature dependency has not been deeply studied in mSCG, however it has been shown that the kinetic of current-opening is increased by the temperature raise generating a small increase of conductance [21]. At 37 °C, the amplitude of the current inhibited by TEA (mainly M-current) is very similar to that inhibited by XE991 in the presence of TEA (mainly TREK-2), indicating a similar influence for these two currents on the resting potential.

### 3.2. Resting Membrane Potential and Excitability

It is well known that I_M_ participates in the control of RMP and excitability in SCG neurons. In fact, I_M_ inhibition depolarizes RMP by about 10 mV and decreases adaptation at room temperature [10]. Our results show that TREK-2 current inhibition has a very mild effect on the RMP at room temperature, but at physiological temperature TREK-2 and M-current inhibitions depolarized the membrane by a similar amount. The minor participation of TREK-2 current on RMP at room temperature should be due to the low activity of these channels at room temperatures and negative voltages [19], however riluzole is still able to hyperpolarize the RMP by about 8 mV at room temperature [6]. Other TREK subfamily members have been shown to be involved in RMP modulation by temperature; for example, in chicken embryonic atrial myocytes, temperature increases from room temperature (20 °C) to physiological temperature (35 °C) elicit a hyperpolarization of 30 mV due to the activation of TREK-1 currents [46].

Most SCG neurons in primary culture present a significant spike frequency adaptation [47], mainly due to I_M_ activation as demonstrated by the inhibition of this current with different substances (Ba^2+^, linopirdine, XE991, Oxo-M) [3,4,10]. Our results show that when the temperature is increased, from room temperature to physiological temperature, the adaptation of SCG neurons is enhanced, probably by the increase of both TREK-2 and M currents. In fact, application of XE991 strongly reduces this adaptation at high temperatures. The increase of temperature also modified spike parameters, such as duration and amplitude of the first spike observed. The reduction of half spike duration by increase of temperature was recovered in the presence of XE991, suggesting that both currents may be implicated in spike repolarization. This is interesting as at room temperature these two currents seem not to contribute to repolarization, and perhaps the increase in the kinetic of activation is responsible for this change in function.

The lack of a selective TREK-2 blocker impairs the study of the contribution of the TREK-2 current to the excitability; in this case, neither riluzole nor TEA are useful to characterize the effect of this current because both drugs can block other currents implicated in the firing [5,42,43]. We attempted to study the implication of the TREK-2 current in the early firing, during the first 60 ms after electrical stimulus, as during this period is when the two first action potentials occur and, at room temperature, I_M_ will be still closed due to its slow activation kinetics [30]. On the contrary, the TREK-2 current should be open at the beginning of the step because it is voltage-independent [31]. Room temperature application of XE991 (3 µM) did not modify either the latency or the frequency of the action potentials, during the first 60 ms after the electrical stimulus application, probably due to the slight activation of TREK-2 currents at room temperature [19] and to the lack of M-current in that time window. When the temperature was increased, both the closing and opening time constants for I_M_ radically decreased from 68 ms (70 ms in rat GCS neurons [40]) to 8 ms and from 157 ms to 7 ms, respectively. This reduction of the time constants means that in the first 60 ms both currents (I_M_ and TREK-2 current) are present at physiological temperature, precluding the study of the implication of the TREK-2 current in the latency and frequency in isolation.

Nevertheless, at physiological temperature we observed a decrease of the latency and an increase of frequency. These parameters are recovered by XE991 application, indicating that activation of M and TREK-2 currents may be involved. In addition, we observed that an increase of temperature reduces the spike duration, an effect also recuperated in the presence of XE991. This reduction could be related to the decrease in latency and the increase in frequency showed. Altogether, we suggest that increase of temperature from room to physiological values increases the amplitude of M and TREK-2 currents and current kinetics.

### 3.3. Physiological Relevance and Conclusion

The study of the importance of M current in maintaining resting potential and neuronal excitability has greatly benefited from the development of two powerful blockers, linopirdine and XE991 [12,29,45]. In this work, we have shown that these two drugs also block the current through the TREK channels, an effect that is particularly important at temperatures close to physiological. These results suggest that in order to understand the complex mechanism underlying the behavior of mSCG neurons, more experiments should be carried out at physiological temperatures and that we must be careful when analyzing the results obtained from the inhibition of the M current, as they can be confused with those obtained from the inhibition of TREK channels.

## 4. Materials and Methods

Animal handling procedures were approved by the Spanish Research Council and the Animal Welfare Committee of the University of Vigo (Code: 07/2014; Date: 24/10/2016), and they were carried out in accordance with Spanish and European directives for the protection of animals used for experimental purposes (RD 05/03/2013; EU 06/03/2010).

### 4.1. Superior Cervical Ganglion (SCG) Culture

Swiss CD-1 mice of both sexes and between 20 and 60 days old were deeply anesthetized using CO2 and sacrificed to remove the SCG. The ganglia were cleaned and digested in collagenase solution for 15 min and in trypsin solution for 30 min. After enzymatic treatments, SCG neurons were disaggregated using a fire-polished Pasteur pipette, centrifuged and plated on laminin (10 µg/mL)-coated dishes with L-15-based culture medium [10,48].

### 4.2. HEK293 Cell Culture and Transfection

HEK293 cell lines were transfected with plasmid pCMV6-AC-GFP (pcDNA 3.1 plasmid) containing mouse gene KCNK10 (TREK-2) using Lipofectamine 3000. Subsequently, HEK293-transfected cell lines were maintained in Dulbecco’s modified Eagle’s medium (DMEM) containing 10% fetal bovine serum (FBS), glucose (4500 μg/mL), sodium pyruvate (110 μg/mL) and L-glutamine (2 mM). After that, the transfected HEK293 cell line was stabilized by adding G418 (500 μg/mL). HEK293 cells were plated onto laminin (10 µg/mL)-coated dishes with DMEM-based culture medium.

### 4.3. Perforated Patch Electrophysiology

One day after the cell culture procedure, we performed the electrophysiological recordings in SCG cells. Patch-clamp protocols were designed using pClamp 10 software (Molecular Devices, Union City, CA, USA) controlling a Digidata 1440A (Molecular Devices) connected to an Axopatch 200B amplifier (Molecular Devices). A heater system (Warner Instruments, Hamden, CT, USA) was used to increase and monitor the temperature. The data were stored and analyzed on a computer using pClamp 10 and Origin 9 software (OriginLab Corporation, Northampton, MA, USA). Whole-cell currents were recorded using the perforated patch technique and amphotericin-B (50–75 µg/mL) was used to gain electrical access to the cell [49,50]. Electrode resistance ranged from 3–6 MΩ, the patches with series resistance >20 MΩ were discarded and junction potentials were not corrected (< 5 mV). The standard bath solution contained (mM): 140 NaCl, 3 KCl, 1 MgCl_2_, 2 CaCl_2_, 10 D-glucose and 10 HEPES (pH was adjusted to 7.2 using Tris [tris(hydroxymethyl)aminomethane]). The standard pipette solution contained (mM): 90 K-acetate, 20 KCl, 3 MgCl_2_, 1 CaCl_2_, 3 EGTA and 40 HEPES (pH was adjusted to 7.2 by adding 20 mM NaOH). The voltage-clamp protocols consisted of gap-free recordings with a holding potential of −30 mV or voltage-jump protocols from −30 mV to −50 mV. Current-clamp protocols consisted of gap-free recordings at a holding membrane potential of −60 mV or the application of one-second current steps from −50 pA to 175 pA every 25 pA. Data were acquired at 2 kHz and filtered using a low-pass filter at 1 kHz or 10 kHz filtering at 5 kHz (for action potentials).

### 4.4. Solutions and Drugs

Drugs were bath-applied during the protocol and some experiments were performed using a drug “cocktail” containing TEA (15 mM), TTX (0.5 µM) and CsCl (1 mM). We used TEA to block the M-currents [1], TTX to block sodium currents and CsCl to inhibit the hyperpolarization-activated cationic H-current [51].

All chemicals were obtained from Sigma-Aldrich (Madrid, Spain), except TTX that was purchased from Tocris Bioscience (Bristol, UK), pCMV6-AC-GFP plasmid belonging to Origene (Rockville, MD, USA) and Lipofectamine 3000 kit from Invitrogen (Life Technologies, California, CA, USA).

### 4.5. Statistics

The data represent the mean±SEM and the statistical significance was determined using a paired or unpaired Student’s t-test in function of the experiment. *p*-values < 0.05 were considered significant.

Linopirdine and XE991 dose–response curves were fitted to sigmoidal curve using a modified Hill equation (Hill1 in Origin8, OriginLab):

(1) $$y=START+\left(END-START\right)\;\frac{x^{n}}{k^{n}+x^{n}}$$

where *START* is the initial value, *END* is the final value, *k* is the Michaelis constant (IC_50_) and *n* is Hill coefficient (slope).

Temperature was enhanced to increase TEA-inhibited current (I_TEA_) and XE991-inhibited current (I_XE991_). Activation curves by temperature were fitted an exponential curve using the following equation,

*y* = *y*_0_ × *A* × *exp*(*R*_0_ × *x*) (2)

where *y*_0_ is the offset, *A* is initial value y *R*_0_ is the ratio. Then, *tau* (63.2% of activated current) was calculated by the following equation:

*tau* = 1/*R*_0_ (3)

The increase rate of previous currents by each 10 °C of temperature (Q10) were calculated using the following equation,

*Q*_10_ = (*R*2/*R*1)^10/(*T*2 − *T*1)^ (4)

where *Q*_10_ is temperature coefficient, *T*2 and *T*1 are two different temperatures, and *R*2 and *R*1 are currents increased at temperatures *T*2 and *T*1.

Action potential parameters were calculated using the first action potential in the first trace showing activity during an IV protocol with the membrane potential clamped at −60 mV. Threshold was estimated by a phase plot of the first derivative of the action potential vs. voltage (dV/dt vs. V). Amplitude was measured from threshold levels to the peak. Action potential duration was measured at half amplitude level.

## Abbreviations

GPCRs	G-protein coupled receptors
I_h_	h-current
I_NaP_	sodium persistent current
I_M_	M-current
I_RIL_	riluzole-activated current
I_TEA_	TEA-inhibited current
I_TREK_	potassium current through TREK channels
I_XE991_	XE991-inhibited current
K2P	two pore domain potassium channels
SCG	superior cervical ganglion
Oxo-M	oxotremorine-M
RMP	resting membrane potential
SFA	spike frequency adaptation
TALK	TWIK-related alkaline pH-activated K^+^ channel
TASK	TWIK-related acid-sensitive K^+^ channel
TEA	tetraethylammonium
THIK	Tandem pore-domain Halothane Inhibited K^+^ channel
TRAAK	TWIK-related arachidonic acid-stimulated K^+^ channel
TREK	TWIK-related K^+^ channel
TRESK	TWIK-related spinal cord K^+^ channel
TTX	tetrodotoxin
TWIK	Tandem of pore-domains in a Weakly Inward rectifying K^+^ channel
